# Dyslipidemia and its predictors among adult workers in eastern Ethiopia: An institution-based cross-sectional study

**DOI:** 10.1371/journal.pone.0291665

**Published:** 2023-10-09

**Authors:** Aboma Motuma, Kasiye Shiferaw, Tesfaye Gobena, Kedir Teji Roba, Yemane Berhane, Alemayehu Worku

**Affiliations:** 1 School of Nursing and Midwifery, College of Health and Medical Sciences, Haramaya University, Harar, Ethiopia; 2 Department of Environmental Health Science, College of Health and Medical Sciences, Haramaya University, Harar, Ethiopia; 3 Department of Epidemiology and Biostatics, Addis Continental Institute of Public Health, Addis Ababa, Ethiopia; 4 Department of Epidemiology and Biostatistics, School of Public Health, Addis Ababa University, Addis Ababa, Ethiopia; Wolkite University, ETHIOPIA

## Abstract

**Introduction:**

Dyslipidemia is a modifiable major risk factor for coronary heart disease. Although, the prevalence of dyslipidemia in high-income countries has been well documented, there is dearth of information about the dyslipidemia among working adults in sub-Saharan African countries including Ethiopia. Therefore, this study aimed to determine the magnitude of dyslipidemia and its associated factors among Haramaya University employees, in Eastern Ethiopia.

**Methods:**

A cross-sectional study was conducted among 1,200 university employees aged 20 to 60 years. Study participants were selected using a simple random sampling method. Data were collected face-to-face interview using a semi-structured questionnaire. Dyslipidemia was defined as unhealthy levels of one or more lipid profile such as high-density lipoprotein, low-density lipoprotein, triglycerides or total cholesterol. Data were entered into Epidata version 3.1 and analyzed using STATA version 16.1 software. Modified Poisson regression with robust variance was used to estimate adjusted prevalence ratios (APR) with its 95% confidence intervals. Statistical significance was declared at P-value < 0.05.

**Results:**

Of 1,164 participants, 59.6% participants had at least one lipid abnormality (i.e., 57.9% among men and 61.5% among women). Of which, 36.8% had high total cholesterol (TC), 21.6% had low high density lipoprotein cholesterol (HDL-c), 22.4% had high low density lipoprotein cholesterol (LDL-c), and 32.6% had high triglyceride (TG). We found that overweight/obesity, sedentary behavior, alcohol consumption, having hypertension and age 45 and above years were significant predictors of dyslipidemia. However, those who served fruit and vegetables more than five per day had significantly reduced prevalence ratio of dyslipidemia.

**Conclusions:**

The high prevalent dyslipidemia among university employees is an important public health problem. Hence, tailored interventions to reduce overweight/obesity, hypertension, alcohol consumption and low fruit and vegetable intake have paramount importance to tackle dyslipidemia particularly among older age.

## Introduction

Dyslipidemia is defined as elevated plasma concentration of LDL cholesterol or triglycerides, or a low plasma HDL cholesterol, total cholesterol or a combination of these features [1, 2]. Major risk factors for cardiovascular disease such as ischemic heart disease and stroke worldwide are attributable to dyslipidemia particularly elevated plasma LDL-cholesterol levels [3]. Dyslipidemia contributed to ∼4.40 million deaths and ∼98.62 million disability-adjusted life years in 2019 [4].

It is still inadequately characterized in African populations despite its major risk factors for cardiovascular disease; an estimated 23.1% of adult Africans (aged ≥25 years) have raised plasma total cholesterol levels [5, 6]. A meta-analysis reported varied prevalence of plasma total cholesterol levels between regions and higher in urban than in rural areas [7]. It is increasing in the general population in Africa, calling for actions to control dyslipidemia [3].

Obesity and overweight are global public health problems; 39% and 13% world population are overweight and obese adults, respectively in 2016 [8]. Dyslipidemia occurs among approximately 60–70% obese patients [9]. Studies across the globe indicated age [10], male sex [10, 11], living in urban areas [10, 11], smoking [11], diabetes [11, 12], overweight [12], obesity [11, 12], central obesity [11, 12], hypertension [11], ethnicity [11], six and above years of work experience [13] and highest socio-economic category [14] were associated with dyslipidemia.

However; there is dearth of information on the association of obesity/overweight and dyslipidemia in Ethiopia, eastern Ethiopia in particular. More research is needed to identify that the combination of dyslipidemia with obesity or hyperglycemia are the most likely combination over time that determines the future status of people [15]. Identifying the magnitude and predictors of dyslipidemia are an important steps for increasing awareness of the problem and for planning health programs properly to prevent its negative clinical effects and may enhance development of biomarkers of obesity-associated dyslipidemia. Further, the authors enquired whether overweight/obesity is associated with dyslipidemia among adult workers. Therefore; the aim of this study was to identify association of obesity with dyslipidemia in the eastern Ethiopia.

## Methods and materials

### Study design and setting

An institutional-based cross-sectional study was conducted to recruit employees to study from December to February, 2019 in the Haramaya University, eastern, Ethiopia. Haramaya University is located at 527km from the capital city, Addis Ababa in the eastern Ethiopia. There are nine colleges, one institute, one referral hospital, one health center, three clinics in the university. Currently, it has 7176 total employees, of which 71.9% are men and 21.1% are women, 78.9% are administrative staff and 21.1% academic staff.

### Population, sample size and sampling technique

All permanent employees in the university were the study population, whereas proportionally selected participants in all departments were study population. Sample size were calculated using single population proportion formula assuming 95% confidence interval, 3% margin of error and 66.7% proportion [16]. Considering 10% non-response rate, the final sample size was 1043, however, we took 1200 which was the largest sample size used for previous studies. Then, simple random sampling method was used to recruit study participants using employees’ payroll as sampling frame. The detail of sample size calculation and sampling methods used were described elsewhere [17].

### Data collection

As it has been elsewhere [17], data were collected using a structured questionnaire adapted from the WHO STEPS through face-to-face interviews and physical measurements, and biochemical tests by trained nurses and laboratory technicians, respectively. The data collection were conducted following: step 1 (interview questions), step 2 (anthropometric measurements) and step 3 (lipid profile) stepwise assessment as explained elsewhere [2, 17]. Show cards pectoral such as the list of work status, tobacco products, alcohol consumption (standard drink), diet (typical fruits and vegetables and serving) and types of physical activities were used to the local context to demonstrate and explain what are meant by some of the questions or items on the instrument.

### Variables and measurements

In this study, the main outcome variable was dyslipidemia. International Diabetics Federation (IDF) criteria [27] consider dyslipidemia if the participants had experienced at least one of the total cholesterol > 200 mg/dl or triglyceride > 150 mg /dl or LDL-c >130 mg/dl or HDL-c < 40mg/dl for male and < 50 mg/dl for female abnormality of lipid parameters above the cut-off value of lipid in the participants’ blood. After face-to-face interview and anthropometric measurement participants were told to skep their breakfast and summoned to come in the early morning the next day at the nearby university’s health center or clinics to give fasting venous blood before work. After eight hours overnight fasting, six milliliters of venous blood was collected from participants in a seating position to measure lipid profile. An appropriate test tube was used (Gel clot activator) to collect the blood sample; serum was separated within 30 minutes of sample collection. The sample was centrifuged at 3000 revolutions per minute for 5 to 10 minutes after retracting the clot and 2 ml pure serum sample was transferred to Nunc tube within 20 minutes. Mindray BS-200 automated chemistry machine was used to analyze sample. We checked the machine using internal quality controls on a daily basis before sample analysis and only after the test passed stated parameters that we proceed to sample analysis. We followed standard infection prevention procedures during blood samples collection from participants.

The digital weighing scale was used to measure weight to the nearest 0.1 kg in light indoor clothing and bare feet or with socks. A portable stadiometer to the nearest 0.1 cm with the participants standing erect posture, without shoes was used to measure height. Body mass index was determined in kilogram/m^2^ as underweight (<18 kg/m^2^), normal (18.5–24.9 kg/m^2^), overweight (25.0–29.5 kg/m^2^), and obese (≥30.0 kg/m^2^) based on WHO definition [18]. Detail description of data collection procedures and variable measurements found in previous publication [17].

### Data quality control

English version questionnaire was translated into the local language (Afan Oromo and Amharic) to make the questionnaire clearer for participants at different levels and translated back to English to check consistence. We reviewed the questionnaire intensively for its content and validity in the context of the study population and pre-test was conducted among 5% of the final sample size at another nearby public university before actual data collection. This enabled us to refine the tool based on the feedback obtained during the pre-test.

An issues such as an interview techniques, anthropometric measurements, precaution that need to be taken during measurements (i.e., height and weight), how to handle sample, sample storage and transportation and standardization were considered during training of data collectors for five days. Further, the data collection process were checked for completeness and accuracy on daily bases by supervisors. A field manual was used during data collection. Calibration of the instruments were checked measuring known weight or length of objects. We used same assigned data collectors to minimize inter-observer variability and maintain consistence of measurements. Data collection was conducted following standard operating procedures. Daily quality control was monitored before running the test for laboratory test.

### Data processing and analysis

Epidata version 3.1 and STATA version 16.1 software were used for the double data entry and analysis, respectively. The data were cleaned and check for missing values and outlier. Descriptive statistic such as mean and standard deviation for continuous data was used to report and proportion was used to describe the categorical data. Dyslipidemia was dichotomized as 1 if participants have abnormality of lipid parameters above the cut-off value and 0 if participants have normal lipid parameters below the cut-off value. The prevalence ratio (PR) using a modified Poisson regression analysis with robust variance estimation model which is preferable for the outcome variable with high prevalence was used to examine associations between dyslipidemia and independent variables. We fitted the backward regression with selected independent variables. Variables with p-value of ≤0.25 in the bivariate analysis transferred to multivariable analysis. Independent variables checked for multi-collinearity before taking them into multivariable models using correlation matrix regression coefficients and variance inflation factors. Model fitness were checked using Akaike’s information criterion. Adjusted PR along with 95% confidence intervals (CIs) were used to report the findings; a p-value of <0.05 was used to declare the statistical significance.

## Results

### Socio-demographic characteristics

From 1200, a total of 1,164 university employees participated in this study (48.6% females and 51.4% males), with a 97% response rate. The major reasons for non-response were absenteeism and tool incompleteness, which is missing completely at random. The majority of the employees, 1721 (58.7%), aged 25–34 years with a mean age of 35.5 (±9.4) years. Nearly two third, 725 (64.9%), respondents were non-office workers, and 739 (63.5%) attended college and above educational status (Table 1).

**Table 1:** Socio-demographic characteristics of Haramaya University employees in Eastern Ethiopia, 2019 (n = 1164).

Socio-demographic variables	Category	Frequency(n)	Percent (%)
Sex	Male	598	51.4
	Female	566	48.6
Age in years	< 24	80	6.9
	25–34	537	46.1
	35–44	324	27.8
	45–54	151	13.0
	≥ 55	72	6.2
Occupation	Non-office worker	755	64.9
	Office worker	409	35.1
Level of education	Primary school (grade1-8)	193	16.6
	Secondary school (grade 9–12)	232	19.9
	College and above (grade 12+)	739	63.5
Monthly salary income in ETB	<2,000	367	31.5
	2000–4000	328	28.2
	4001–6000	168	14.4
	> 6000	301	25.9
Ethnicity	Oromo	509	43.7
	Amhara	549	47.2
	Others^a^	106	9.1
Marital status	Never married	427	36.7
	Married	667	57.3
	Divorced/widowed	70	6.0
Religion	Orthodox	722	62.0
	Muslim	219	18.8
	Protestant	197	16.9
	Others^b^	26	2.3

a: Gurage, Tigraway, Harari, Wolaita

b: Catholic, Traditional; ETB: Ethiopia Birr

Of 1,164 study participants, 36.8% had high total cholesterol, 21.6% had low HDL-C, 22.4% had high LDL-C, and 32.6% had high TG. Of these measures, at least one lipid abnormality was diagnosed in 57.9% of men and 61.5% of women. The overall proportion of dyslipidemia among university employees in the study area was 59.6% (95% CI = 56.7%–62.5%) **(Table 2)**.

**Table 2:** Biochemical characteristics of study participants among Haramaya University Employees, Eastern of Ethiopia, 2019 (n = 1,164).

Variable	Category	Frequency	Percent
LD-c	Normal (<130 mg/dl)	903	77.6
	High (≥ 130 mg/dl	261	22.4
Total cholesterol	Normal (<200 mg/dl)	736	63.2
	High (≥ 200 mg/dl)	428	36.8
HDL-c	Desirable (≥ 50 mg/ dl)	912	78.4
	Low (< 50 mg /dl)	252	21.6
Triglycerides	Normal (<150 mg /dl)	785	67.4
	High (≥ 150 mg/dl)	379	32.6
Waist to hip ratio	Normal	790	67.9
	High	374	32.1
Fast blood sugar	Normal(<100 mg/dl)	934	80.2
	High (≥ 100 mg/dl)	230	19.8
Dyslipidemia	Yes	694	59.6
	No	470	40.4

### Components of the abnormal dyslipidemia

**Fig 1:**
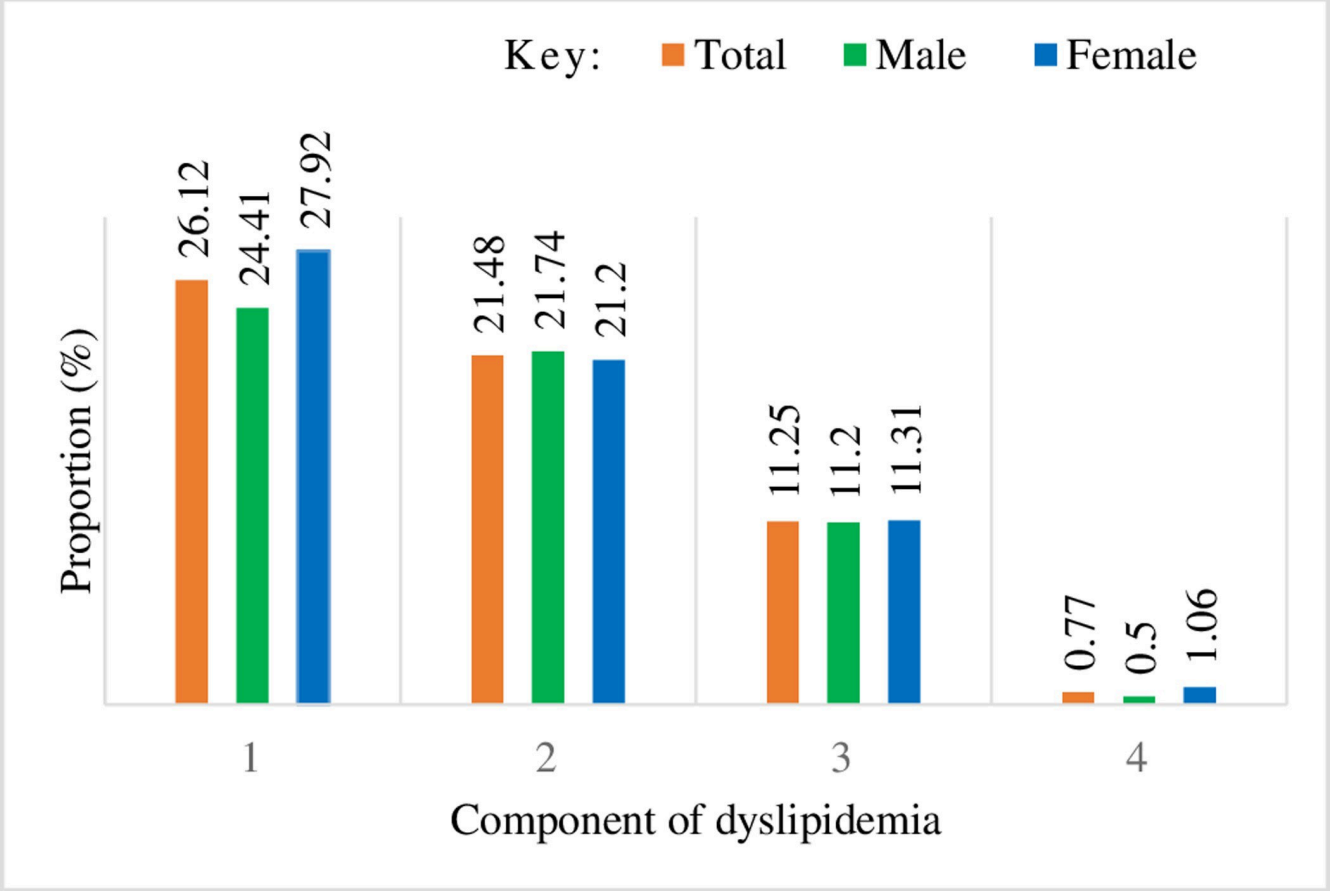
Distribution of dyslipidemia component(s) by sex among adult workers in eastern Ethiopia.

Among study participants, 304 (21.2%) had only one components of lipid abnormality, 390 (33.6%) had at least 2 or above lipid abnormality (Fig 1).

### Participants’ behavioral characteristics

One hundred-thirty one respondents (11.3%) reported having a history of smoking cigarettes; of which 5.1% were current smokers. Half of respondents, 561 (48.2%) drank alcohol in the last one year. Almost all study participants, 1108(95.2%) had at least one serving of fruit and vegetable per day. Four hundred thirteen (35.5%) and 571 (49.1%) participants had overweight or obesity and low physical activity, respectively **(Table 3)**.

**Table 3:** Lifestyle characteristics of Haramaya University employees, eastern Ethiopia, 2019 (n = 1,164).

Life style	Category	Frequency	Percent
*Khat* chewing	Yes	396	34.0
	No	768	66.0
Physical activity level	Low (≤600 MET min/week)	571	49.1
	Moderate (600–2999 MET min/week)	367	31.5
	High (≥3000 MET min/week)	226	19.4
Smoking status	Yes	131	11.3
	No	1033	88.7
Alcohol consumption	Yes	561	48.2
	No	603	51.8
Serving Fruit and vegetable	0 servings per day	56	4.8
	At least one-time servings per day	1108	95.2
Sedentary behavior	< 8 hours per day	928	79.7
	≥ 8 hours per day	236	20.3
Body mass index	Underweight	113	9.7
	Normal weight	638	54.8
	Overweight	305	26.2
	Obesity	108	9.3

### Factors associated with dyslipidemia

In bivariate analysis variables like age, sex, marital status, educational level, monthly income, physical inactivity, low serving of fruit and vegetables, body mass index, alcohol consumption, and having diabetes, *khat* chewing, sedentary behavior, consumption of fruits and self-report of health status had a p-value of < 0.25; hence, selected as candidate variables for multivariable Poisson regression with robust variance analysis. In multivariable analysis, age, body mass index, serving of fruit and vegetable, alcohol consumption, being hypertensive and sedentary behavior persisted to be statistically significant variables associated with dyslipidemia at a p-value of < 0.05.

The prevalence ratio of dyslipidemia were 1.3 and 1.4 times higher among workers aged 45–54 (APR = 1.29, 95% CI: 1.02–1.62) and 55 years and older (APR = 1.42, 95% CI: 1.12–1.79), respectively compared to those who aged 24 years and younger. Besides, the prevalence ratio of dyslipidemia were 1.5 (APR = 1.54, 95% CI 1.27–1.95) and 1.4 (APR = 1.42, 95% CI: 1.27–2.09) times higher among individuals who are overweight and obese, respectively compared to their underweight counterpart. Respondents having sedentary behavior ≥ 8 hours per day had 1.1 times higher prevalence ratio of dyslipidemia compared to their counterparts (APR = 1.11, 95% CI: 1.01–1.22). Hypertensive participants had 1.2 times higher the prevalence of dyslipidemia (APR = 1.17, 95% CI: 1.07–1.28) than non-hypertensive. Respondents who consume alcohol 5–7 days per week had 1.2 higher prevalence of dyslipidemia as compared to their counterparts (APR = 1.18, 95% CI: 1.05–1.32). On the other hand, the prevalence ratio of dyslipidemia were 29% lower among individuals with higher consumption of fruits and vegetables compared to those with lower consumption of fruits and vegetables (APR = 0.71,95% CI:0.55–0.92) **(Table 4)**.

**Table 4:** Crude and adjusted analysis of factors associated with dyslipidemia among Haramaya University employees, Eastern Ethiopia, 2019.

Characteristics	Dyslipidemia		CPR (95% CI)	APR (95% CI)	P-value
	Yes (%)	No (%)			
Sex					
Male	346 (57.9)	252 (42.1)	1	1	
Female	348(61.5)	218 (38.5)	1.06(0.97–1.17)	1.04(0.94–1.14)	0.468
Age group					
<24 years	41 (51.2)	39 (48.8)	1	1	
25–34 years	238 (44.3)	299 (55.7)	0.86(0.68–1.09)	0.82(0.65–1.03)	0.092
35–44 years	228 (70.4)	96(29.6)	1.37(1.10–1.72)*	1.18(0.94–1.48)	0.153
45–54 years	123(81.5)	28(18.5)	1.59(1.27–1.99)*	1.29(1.02–1.62)*	0.035
≥ 55 years	64 (88.9)	8(11.1)	1.73(1.38–2.18)*	1.42(1.12–1.79)*	0.009
BMI					
Underweight	43 (38.1)	70 (62.9)	1	1	
Normal	345(54.1)	293 (45.9)	1.42(1.11–1.82)*	1.34(1.06–1.70)*	0.013
Overweight	215 (70.5)	90 (29.5)	1.85(1.45–2.37)*	1.54(1.22–1.95)*	0.000
Obese	91(84.3)	17(15.7)	2.21(1.73–2.84)*	1.42(1.27–2.09)*	0.000
Serving fruits and vegetables					
None serving	46(82.1)	10 (17.9)	1	1	
1–2 serving per day	441(59.8)	291 (40.2)	0.73(0.64–0.83)*	0.86(0.75–0.98)*	0.029
3–4 serving per day	165(59.6)	112(40.4)	0.72(0.62–0.85)*	0.87(0.75–1.02)	0.084
≥ 5 serving per day	42(45.2)	51(54.8)	0.55(0.43–0.71)*	0.71(0.55–0.92)*	0.009
Sedentary behavior					
< 8 hours per day	519(55.9)	409(44.1)	1	1	
≥ 8 hours per day	175(74.2)	61(25.8)	1.33(1.21–1.46)*	1.11(1.01–1.22)*	0.035
Alcohol intake					
< once day/month	506(51.7)	381(42.9)	1	1	
1–4 days/week	138(63.0)	81(37.0)	1.11(0.98–1.24)	1.09(0.97–1.22)	0.142
5–7 days/week	50(86.2)	8(13.8)	1.51(1.34–1.70)*	1.18(1.05–1.32)*	0.005
Hypertensive					
No	472(54.0)	402(46.0)	1	1	
Yes	222(76.5)	68 (23.5)	1.30(1.30–1.55)*	1.17(1.07–1.28)*	0.001

*p-value is significant or less than 0.05.

## Discussion

This study identify the magnitude of dyslipidemia and its association with overweight/obesity among university employees in the eastern Ethiopia. Dyslipidemia is a prevalent public health problem; age, body mass index, serving fruit and vegetable, sedentary behavior, alcohol intake and hypertension were significantly associated with dyslipidemia.

The study revealed that 60% of university employees had dyslipidemia. Its prevalence is 88% among advanced age Columbian population [19] and 66.7% among study subjects in Mekele city, Ethiopia [16]. An older age (mean age 50.7 years) is associated with prevalent dyslipidemia unlike our study among working adult (18 to 60 year old, mean age 35 years). On the other hand, meta-analysis in Africa revealed 25.5% of adult population had dyslipidemia [7]. Similarly, studies in Korea and China indicated 32.6% and 43% of participants had dyslipidemia, respectively [12, 20]. The meta-analysis conducted in Africa, studies in Korea and china are population-based studies. Some of the cutoffs used are higher than the limits that have been agreed on and varying definition of dyslipidemia may contributed to lower prevalence. For instance, Chinese guideline defined dyslipidemia as having one or more condition; TC, LDL-C, HDL-C, TG or if clients were taking anti-dyslipidemia medication.

Age increment is associated with increased prevalence ratio of dyslipidemia. Being above 64 years and 40–64 years old were identified as significant predictors of dyslipidemia [16]. Similarly, study in Yemeni university echoed this finding [10]. Study in Asia and Pacific Region revealed levels of serum lipids change with age [21]. The finding highlights older age need tailored interventions to reduce dyslipidemia at this age.

More importantly, participants who are overweight or obese have increased prevalence ratio of dyslipidemia. Mekele city’s study in Ethiopia, indicated significant association of overweight and obesity with dyslipidemia [16]. Obesity was independently associated with hypercholesterolemia and hypertriglyceridemia in Jordan [22]. Further, obesity and central obesity were positively correlated with dyslipidemia in china [11]. Similarly, study in rural and urban china indicated overweight, obesity, and central obesity was associated with dyslipidemia [12]. Obesity, metabolic syndrome and dyslipidemia may be linked to intravascular lipolysis, anti-lipolytic effect of insulin, adipose tissue resistance and insulin resistance in peripheral tissues leading to an enhanced hepatic flux of fatty acids from dietary sources [23] and significantly associated with myocardial infarction risk among men [6].

Hypertension were significantly associated with dyslipidemia. The odds of hypertension increased among participants who had dyslipidemia Southwest Ethiopia [24]. Hypertension was independently associated with hypercholesterolemia and hypertriglyceridemia [22]. Study in china also indicated hypertension was correlated with dyslipidemia [11]. This implies controlling hypertension play vital role to reduce dyslipidemia or vice versa.

Participants who are serving fruit and vegetable greater than or equal to five serving per day had decreased prevalence ratio of dyslipidemia. Participants who consumed for 6–7 days per week had lower risks of high dyslipidemia, compared to fruit consumption of 0–2 days per week [25]. Similarly, study call for strategies stimulate health diets habits [14]. By contrast, a higher median daily intake of vegetables is reported by participants with dyslipidemia compared to those without dyslipidemia [26].

Similarly, sedentary behavior is associated with increased prevalence ratio of dyslipidemia. Study call for strategies stimulate a physically active lifestyle to minimize health problems [14]. Further, walking <150 minutes per week was identified as significant predictors of dyslipidemia in Mekele City, Northern Ethiopia [16]. Moderate physical activities decreased the odds of dyslipidemia in Jordan [27]. Study in Wuhan city indicated sedentary behavior increased odds of dyslipidemia; whereas, dyslipidemia is linked to physical inactivity [28]. Hence, life style modification such as reducing physical inactivity, specific behavior change techniques across different contexts and settings are vital to reduce dyslipidemia.

Dyslipidemia is attributable to alcohol intake. Dyslipidemia is higher among participants with higher intake of refined grains and sugar-sweetened beverages [26]. However, study in china revealed alcohol consumption was linked to lower risk of dyslipidemia [11]. According to Korean Genome and Epidemiology Study, alcohol consumption was inversely associated with low HDL-C and dyslipidemia particularly among female [29]. This highlights further study employing appropriate design to establish causal relationship of alcohol consumption and dyslipidemia and its pathophysiology.

### Strength and limitation of the study

A large sample size and use of standardized data collection tools (WHO STEPwise approach) were the strengths of this study. This study was conducted with the highest precautions during anthropometric data collections, equipment calibration, and standardization of procedure to minimize errors. Moreover, this study was the first of its kind among university employees in Ethiopia and can be fairly generalized for this category of participants in areas where there are contextual working adults. However, this study was subject to certain limitations, such as social desirability bias, that may affect the reporting of lifestyle habits (smoking, *khat* chewing, and alcohol consumption) as they are socially unacceptable in the university community. The results of this study must be interpreted with caution as the study population was drawn only from one institution.

Lastly, we were unable to declare causal relationship between explanatory and outcome variables since our study cross-sectional study.

## Conclusion

Dyslipidemia is prevalent public health problem among university employees in the eastern Ethiopia. This study highlights reduction of obesity, sedentary behaviors and alcohol intake modifiable associated factors to reduce dyslipidemia. Further, physically active life style and serving fruit and vegetables and controlling hypertension alleviate dyslipidemia. An effort to reduce CVDs in Ethiopia should integrate effective dyslipidemia detection and treatment as well as life style change and obesity reduction.

## Supporting information

S1 Data (DTA)
